# Case Report: Sellar hemangioblastoma mimicking a pituitary macroadenoma: a diagnostic and surgical pitfall

**DOI:** 10.3389/fonc.2026.1735533

**Published:** 2026-03-25

**Authors:** Jie Li, Fei Xie, Jia Nie, Xingchen Li

**Affiliations:** 1 Department of Radiology, Deyang People’s Hospital, Deyang, Sichuan, China; 2 Department of Oncology, Deyang People’s Hospital, Deyang, Sichuan, China; 3 Department of Pathology, Deyang People’s Hospital, Deyang, Sichuan, China

**Keywords:** differential diagnosis, hemangioblastoma, intraoperative hemorrhage, pituitary adenoma, sellar

## Abstract

**Background:**

Sellar hemangioblastoma (HBL), an exceedingly rare and hypervascular tumor, represents a critical diagnostic pitfall as it frequently mimics the radiological features of a common pituitary adenoma. This misidentification poses a substantial risk of unexpected and severe intraoperative hemorrhage, complicating surgical management.

**Case presentation:**

A 64-year-old female presented with intermittent dizziness. Magnetic resonance imaging revealed a large, avidly enhancing solid sellar mass with cystic changes, which was preoperatively diagnosed as a pituitary macroadenoma. However, an attempted endoscopic endonasal resection was complicated by profuse, difficult-to-control hemorrhage, resulting in only a subtotal resection. The definitive diagnosis, established by postoperative histopathology, was hemangioblastoma (WHO Grade I). A retrospective review of the imaging highlighted key features suggestive of HBL, including prominent vascular flow voids and avid early-phase arterial enhancement, which were initially overlooked due to diagnostic bias toward the more common pathology.

**Conclusions:**

This case underscores the critical importance of maintaining a high index of suspicion for HBL when evaluating any sellar mass, despite its rarity. Radiological “red flags” such as prominent flow voids and early arterial enhancement on dynamic imaging should prompt both clinicians and radiologists to include HBL in the differential diagnosis. Accurate preoperative identification is paramount for optimizing surgical strategy—including consideration of preoperative embolization and the surgical approach—to mitigate the risk of life-threatening hemorrhage. Furthermore, suspected sellar HBL should trigger an evaluation for Von Hippel-Lindau (VHL) disease, irrespective of symptoms, as confirming this diagnosis may completely reshape the treatment strategy.

## Introduction

Hemangioblastomas (HBLs) are benign, slow-growing vascular tumors of the central nervous system (CNS), classified as World Health Organization (WHO) Grade I. They account for only 1% to 2.5% of all intracranial tumors, but this incidence rises to 7% to 12% of primary tumors in the posterior fossa (1, 2). The most common sites of occurrence are the cerebellar hemispheres, brainstem, and spinal cord (3). Supratentorial HBLs are exceedingly rare, comprising only 1%-6% of all CNS HBLs. Although the sellar/suprasellar region is a primary site for supratentorial HBLs (accounting for 26%-28% of such cases), their extreme overall rarity in this location makes them a significant diagnostic challenge (4, 5).

The sellar region possesses a complex anatomy, where pituitary adenomas are the most prevalent space-occupying lesions (6). Consequently, a mass in this location is often presumptively diagnosed as a pituitary adenoma. This assumption, however, carries substantial risk. HBLs are characteristically hypervascular tumors composed of a dense network of thin-walled, closely packed capillaries interspersed with lipid-laden stromal cells. This distinct histological architecture manifests radiologically as avid enhancement and prominent serpentine flow voids (7). In contrast, pituitary adenomas typically lack such extreme vascularity. Misdiagnosing an HBL as an adenoma can therefore lead to unexpected, severe intraoperative hemorrhage, complicating surgical resection and significantly increasing the risk of patient morbidity.

Herein, we report the case of a 64-year-old female with a sellar HBL that was preoperatively misdiagnosed as a pituitary macroadenoma. We aim to describe its clinical, radiological, and pathological features in detail. Furthermore, through a review of the literature, we will focus on its imaging characteristics and key differential diagnostic points, with the objective of improving the accuracy of preoperative identification and optimizing surgical strategies.

## Case presentation

A 64-year-old female presented to our institution with a chief complaint of intermittent dizziness persisting for over one year. The patient described the sensation as non-rotatory lightheadedness, which occurred without obvious triggers and was not exacerbated by postural changes. She denied experiencing true vertigo (spinning sensation) or subjective visual disturbances, such as blurred vision or diplopia. A non-contrast computed tomography (CT) scan performed at an outside hospital had indicated a sellar mass, prompting her admission for further evaluation.

**Figure 1:**
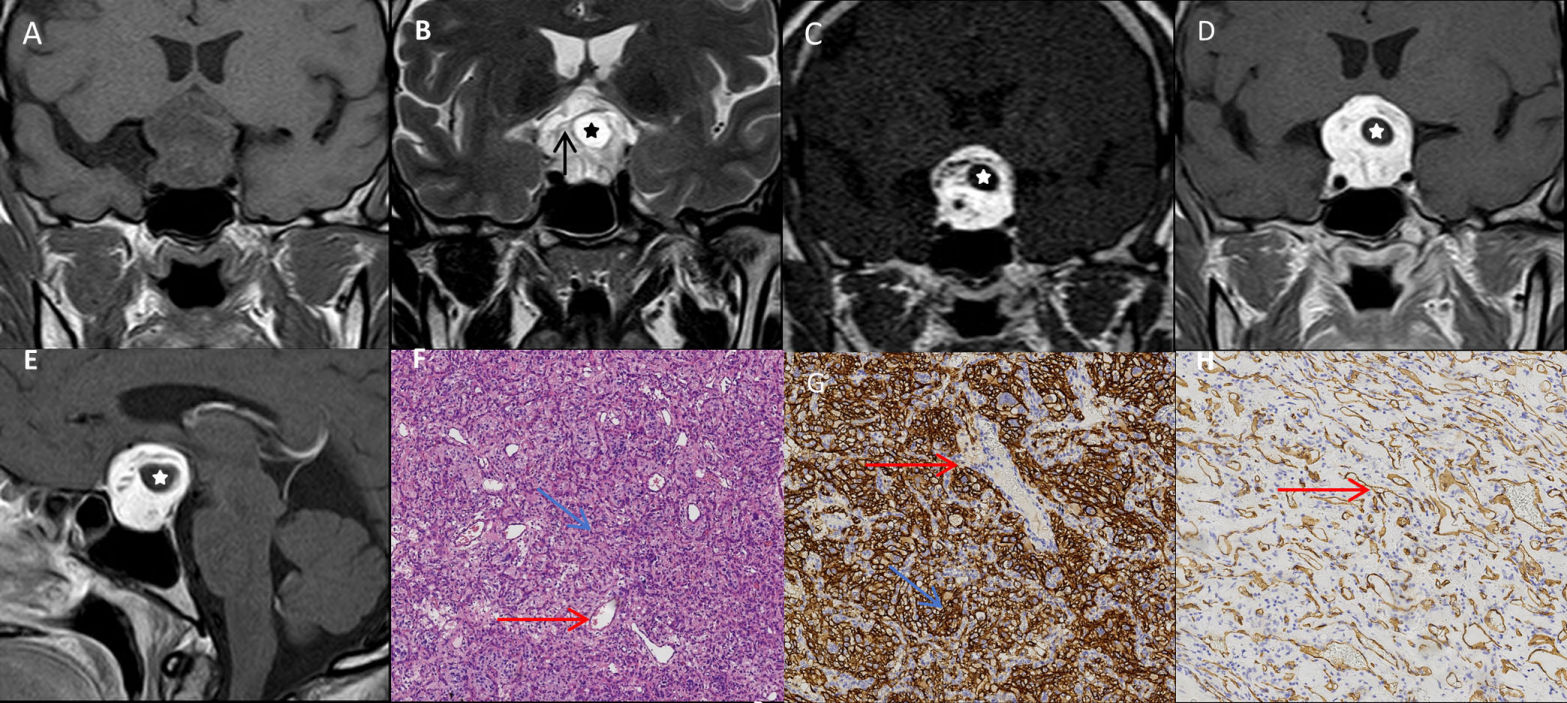
Preoperative MRI findings **(A-E)** and pathological examinations **(F-H)**: **(A)** The coronal T1-weighted image reveals a sellar-suprasellar mass displaying mixed iso- and hypointense signals. **(B)** The coronal T2-weighted image demonstrates heterogeneous hyperintense signals with cystic changes (asterisk) and vascular flow voids (black arrow), showing compression of the optic chiasm, mild bilateral cavernous sinus narrowing, and partial encasement of the internal carotid arteries. **(C)** Dynamic contrast-enhanced imaging shows marked heterogeneous enhancement with tortuous and cystic non-enhancing structures (asterisk) in the early arterial phase. **(D, E)** This is followed by persistent homogeneous enhancement (excluding cystic areas) (asterisk) in the delayed phase. **(F)** Microscopy showed sheets of short spindle tumor cells with lipid-rich cytoplasm (blue arrow), abundant capillaries (red arrows), and rich stromal reticulin fibers. **(G, H)** The immunohistochemistry demonstrated diffuse CD56 positivity (blue arrow), CD34-positive capillaries(red arrows) (tumor cells negative), NSE positivity, focal S-100 reactivity, and rare Ki-67-positive cells.

Upon admission to our institution, a pituitary magnetic resonance imaging (MRI) was performed. It revealed a well-demarcated sellar and suprasellar mass, measuring approximately 2.6 × 3.3 × 2.6 cm. The lesion appeared as a mixed iso- and hypointense signal on T1-weighted imaging (**Figure 1A**) and heterogeneous high signal intensity on T2-weighted imaging, with cystic degeneration and multiple serpentine flow voids. The mass compressed the optic chiasm superiorly, caused mild compression of both cavernous sinuses, and partially encased the internal carotid arteries (**Figure 1B**). Dynamic contrast-enhanced imaging demonstrated marked, heterogeneous enhancement in the early arterial phase, which became more homogeneous in the delayed phase; the cystic areas remained non-enhancing (**Figures 1C–E**).

Based on the mass effect observed on imaging, a detailed physical examination, including neuro-ophthalmological evaluation, was conducted, which revealed no visual acuity deficits or visual field defects. Furthermore, a comprehensive endocrine evaluation of the pituitary-target gland axes was performed. Plasma levels of anterior pituitary hormones—including adrenocorticotropic hormone (ACTH), growth hormone (GH), follicle-stimulating hormone (FSH), luteinizing hormone (LH), and prolactin (PRL)—as well as corresponding target gland hormones (serum cortisol, estradiol, and progesterone) were all within normal reference ranges. Consequently, a presumptive diagnosis of non-functioning pituitary macroadenoma with cystic degeneration was made.

The patient subsequently underwent a neuro-navigation-assisted endoscopic endonasal approach with the planned intent of gross total resection. Given the high preoperative confidence in the diagnosis of a pituitary macroadenoma, routine intraoperative frozen section was not initially performed. However, the procedure was complicated by unexpected, profuse hemorrhage upon tumor manipulation with a suction device and curette. The surgical priority immediately shifted from resection to hemostasis. Bleeding was eventually controlled using gelatin sponges and a flowable gelatin matrix. At this critical juncture, obtaining a frozen section was deemed unsafe, as waiting for pathology would have prolonged operative time and blood loss without altering the necessity to abort the resection. Consequently, the surgery was terminated with subtotal removal of the tumor.

The resected specimen was sent for histopathological examination. Microscopy revealed sheets of short spindle-shaped tumor cells with lipid-rich cytoplasm, embedded within a dense capillary network and abundant stromal reticulin fibers (**Figure 1F**). Immunohistochemical staining demonstrated diffuse CD56 positivity, CD34-positive capillaries (tumor cells negative), NSE positivity, focal S-100 reactivity, and rare Ki-67-positive cells (**Figures 1G, H**). Collectively, these findings established the definitive diagnosis of hemangioblastoma (HBL, WHO Grade I).

**Figure 2:**
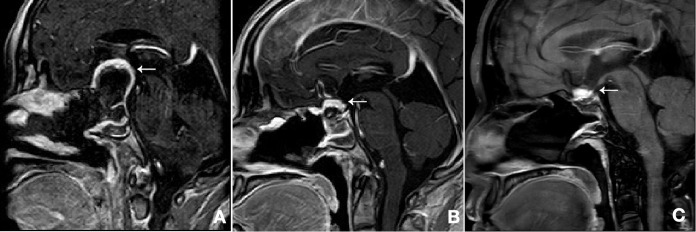
Postoperative and follow-up magnetic resonance imaging: Serial contrast-enhanced T1-weighted sagittal images demonstrate the persistence of the residual tumor (arrow) on postoperative day 6 **(A)**, at the 5-month follow-up **(B)**, and at the 1-year follow-up **(C)**. Across the scans, the tumor’s volume remained stable. Notably, observed alterations in the residual tumor’s morphology are attributed to the expected evolution of the surgical bed and absorption of hemostatic material, rather than true tumor dynamics.

The postoperative course was uneventful. A baseline postoperative MRI on day 6 confirmed a curvilinear residual tumor with avid enhancement in the surgical bed (**Figure 2A**). Given the strong association with Von Hippel-Lindau (VHL) disease, genetic and systemic screening was recommended; however, the patient declined this workup and was discharged in good condition.

The patient was followed up at 5 months and subsequently at one year postoperatively. Clinically, she remained in good condition, with complete resolution of her presenting dizziness. Serial neuro-ophthalmological evaluations showed stable visual acuity and full visual fields bilaterally throughout the follow-up period. Furthermore, she reported no new symptoms suggestive of pituitary dysfunction. Serial follow-up MRIs confirmed the persistence of the residual tumor. Notably, there was no significant interval change in its volume at either the 5-month or 1-year mark (**Figures 2B, C**).

## Discussion

This case highlights a critical diagnostic pitfall: the misidentification of a rare sellar HBL as a common pituitary macroadenoma. Although flow voids and avid early enhancement are well-established radiographic hallmarks of HBLs (8, 9), their diagnostic significance in the sellar region is often underestimated due to the overwhelming prevalence of pituitary adenomas. This statistical disparity creates a strong “anchoring bias.” Consequently, subtle but critical signs, such as intratumoral flow voids, may be erroneously rationalized as displaced normal vessels rather than triggering a search for a hypervascular neoplasm. Such cognitive shortcuts, however, carry substantial risks. As demonstrated in our case and others (10–12), failure to correctly interpret these signs leads to unexpected, torrential intraoperative hemorrhage, complicating resection and endangering patient safety. Therefore, recognizing that anatomical probability should not supersede morphological evidence is crucial for preventing such surgical complications.

This bias is exacerbated by striking imaging similarities. Indeed, HBLs can manifest in six characteristic imaging patterns: large cyst with a small solid nodule (the most common and classic type), large cyst with a large solid component, microcystic with a large solid component, microcystic with a small solid nodule, purely cystic, and purely solid—with the purely solid type being the most radiologically atypical (13). Solid HBLs typically appear as heterogeneous hypo- to isointense on T1-weighted imaging (T1WI) and hyperintense on T2-weighted imaging (T2WI), accompanied by characteristic punctate or serpentine vascular flow voids, frequent intratumoral necrosis, marked enhancement of the solid component, and significant peritumoral edema (14). However, unlike intra-axial lesions, our sellar HBL lacked significant parenchymal edema, likely due to its extra-axial expansion into the cisterns rather than invasion of the brain tissue. Consistent with other solid variants, our case demonstrated characteristic intratumoral cystic changes, multiple vascular flow voids, and avid enhancement of its solid component. While intratumoral cysts can represent dilated vascular spaces or necrotic regions (7), the cystic changes in our case are most consistent with post-necrotic resorption, given the absence of both flow voids and contrast enhancement in these areas. Notably, while the cystic type with a mural nodule is classic, a review of published sellar HBL cases reveals that solid or predominantly solid mixed types are a common presentation in this region (10, 15, 16). The features of our case are highly consistent with these reports. These radiological signs reflect the tumor’s hypervascular pathological basis, which is related to its histological composition of abundant vascular channels and stromal cells (17). It was precisely this highly vascular nature that led to the difficult-to-control hemorrhage during surgery in our case, thereby highlighting the critical value of these imaging features in preoperative risk assessment.

To fully leverage the value of these imaging features for preoperative risk assessment, a careful differential diagnosis with other major sellar lesions is imperative. First, as the most common lesion in this region, pituitary macroadenomas have a fundamentally different enhancement pattern from HBLs. Although the degree of enhancement in pituitary adenomas can vary with their consistency (soft, firm, or intermediate), with even firm adenomas showing marked enhancement, a common principle is that their enhancement phase is typically delayed compared to the normal pituitary gland and infundibulum (18). This is in stark contrast to HBLs, which demonstrate avid enhancement in the early arterial phase, often even earlier than the infundibulum (19). This temporal difference in enhancement is a core differentiating point and aligns perfectly with the imaging findings in our case. Second, craniopharyngiomas are primarily identified by their characteristic calcifications, which are absent in HBLs (15, 16). Third, meningiomas, particularly hypervascular types, can be distinguished from HBLs by their typical “dural tail sign,” a feature usually lacking in sellar HBLs (20).

Beyond these common entities, the differential diagnosis must extend to rarer lesions, especially those that can mimic the hypervascular nature of HBL. Foremost among these are Solitary Fibrous Tumors (SFT), which, according to the 2021 WHO classification, encompass a spectrum from grade 1 to grades 2 and 3—the latter corresponding to the obsolete terms “hemangiopericytoma” and “anaplastic hemangiopericytoma” (21). Like HBLs, SFTs are hypervascular lesions that typically exhibit avid early arterial enhancement and prominent flow voids (22). Notably, intratumoral necrosis is a hallmark of WHO grade 3 SFTs (23). Therefore, given the significant necrotic areas observed in our case, differentiation from a WHO grade 3 SFT is particularly critical. However, high-grade SFTs are generally more aggressive, frequently demonstrating invasive margins and even erosion of adjacent bone structures (24, 25). Moreover, SFTs almost invariably display a close relationship with the dura mater (24)—a feature that can help distinguish them from our case. Furthermore, rare cystic variants of SFTs must be differentiated from cystic HBLs; however, cystic SFTs tend to show marked enhancement of the cyst wall itself without a distinct mural nodule (25). Intrasellar cavernous hemangiomas also show marked enhancement, but it is typically progressive and appears as markedly hyperintense on T2-weighted imaging. Moreover, they often possess a dural pseudocapsule that can simulate a meningioma’s “dural tail sign” (26). Lastly, germ cell tumors, predominantly affecting children and adolescents, characteristically involve the suprasellar region with inferior extension into the sella to primarily involve the posterior pituitary. The consequent loss of the posterior pituitary’s normal T1-hyperintense signal is their most reliable diagnostic sign and a key feature for differentiation from HBLs (27).

Accurate differentiation is critical because the management of HBL differs fundamentally from that of other sellar lesions. The clinical course of this case highlights the risks of misdiagnosis. The principle of treatment for sellar HBL is complete surgical resection (15). Due to the tumor’s extreme vascularity, many authors advocate for a craniotomy approach, as a transsphenoidal route may lead to uncontrollable hemorrhage, potentially only allowing for a biopsy or subtotal resection (11, 28). The massive hemorrhage encountered in our case, which utilized an endoscopic endonasal approach, corroborates this risk. Therefore, a high preoperative suspicion of HBL can guide the surgical team in formulating a more comprehensive plan. This includes performing Digital Subtraction Angiography (DSA) or Computed Tomography Angiography (CTA) to identify feeding arteries (29) and to evaluate the feasibility of preoperative embolization. Preoperative embolization has been proven effective in reducing tumor hypervascularity and intraoperative blood loss, creating favorable conditions for safe, total resection (30, 31). In summary, for suspected hypervascular sellar tumors, meticulous preoperative planning is essential. Vascular assessment and the consideration of preoperative embolization should be individualized, balancing the potential benefits of reduced intraoperative bleeding against the inherent risks of the embolization procedure itself.

Furthermore, the strong association between sellar HBLs and VHL disease—60-84% compared to 20-40% in other locations (5, 20, 32) —has critical implications for preoperative strategy.

When imaging features such as flow voids and early enhancement raise suspicion of a hemangioblastoma, validating a VHL diagnosis is paramount as it opens the door to non-surgical management. Recently, belzutifan, a specific HIF-2α inhibitor, has been approved for VHL-associated CNS hemangioblastomas (33). By targeting the dysregulated hypoxia pathway intrinsic to VHL-mutated tumors, this agent offers a potent alternative to high-risk surgery. In the pivotal open-label trial involving VHL patients, the subgroup with measurable CNS hemangioblastomas achieved an objective response rate of 63% (34). Consistent with these findings, recent case reports have further documented its efficacy in shrinking or stabilizing such tumors in complex clinical scenarios (35, 36).

In our case, a higher preoperative index of suspicion could have prompted genetic testing. If a VHL mutation had been identified, the treatment plan might have been fundamentally altered: instead of an endoscopic resection complicated by massive hemorrhage, the patient could have been considered for upfront belzutifan therapy. This possibility suggests that for sellar lesions radiographically suspected to be hemangioblastomas, genetic profiling should be viewed as an integral part of the preoperative assessment. Furthermore, recent success in treating a sporadic case (37) suggests that belzutifan may offer a salvage avenue even for patients without a confirmed germline mutation, although the reliability of this expanded strategy awaits confirmation in prospective trials.

This study has several limitations. First, as a single case report, the generalizability of its findings is limited. Second, because the patient refused systematic screening for VHL disease, we could not definitively classify this case as sporadic or assess for other potential VHL-related lesions, rendering the case information incomplete. Finally, the follow-up period for this patient was relatively short. Therefore, the long-term natural history of the residual tumor and the growth behavior following subtotal resection remain to be observed.

## Conclusion

Sellar HBL is a rare diagnostic pitfall, as its imaging findings often mimic those of a pituitary adenoma, leading to a risk of unexpected intraoperative hemorrhage. This case underscores that despite the rarity of HBL, clinicians should maintain a high index of suspicion when a sellar mass demonstrates prominent vascular flow voids and early arterial phase enhancement on MRI. Preoperative identification of these hypervascular features is critical for formulating safer surgical strategies, including assessing the necessity of preoperative embolization and selecting the optimal surgical approach to minimize hemorrhagic risk. Furthermore, once the diagnosis is suspected, it should trigger an evaluation for Von Hippel-Lindau (VHL) disease, including genetic counseling and systemic screening irrespective of symptoms, since establishing this diagnosis may fundamentally alter the treatment strategy.

## Data Availability

The original contributions presented in the study are included in the article/supplementary material. Further inquiries can be directed to the corresponding author.
